# Effects of Viscosity Modification on Sensory Perception, Hedonic Evaluation, and Expected Fullness of Whey-Based High-Protein Beverages Under Blind and Informed Conditions

**DOI:** 10.3390/foods15162871

**Published:** 2026-08-17

**Authors:** Neswa Neivia Saeeda, Callista Medina, Dwi Larasatie Nur Fibri, Akira Ishida

**Affiliations:** 1 Graduate School of Agricultural Science, Kobe University, Kobe 657-8501, Japan; neswaneivia11@gmail.com; 2 Department of Food and Agricultural Product Technology, Faculty of Agricultural Technology, Universitas Gadjah Mada, Jl. Flora, Bulaksumur, Sleman 55281, D.I. Yogyakarta, Indonesia; callistcallista@gmail.com; 3 Center of Excellence for Indonesian Gastronomy, Jl. Flora, Bulaksumur, Sleman 55281, D.I. Yogyakarta, Indonesia

**Keywords:** whey-based high-protein beverages, viscosity, expected fullness, sensory evaluation, Linear Mixed Models

## Abstract

This study investigated the effects of xanthan-gum-induced viscosity on sensory perception, expected fullness, and panelists’ acceptance of whey-based high-protein beverages under sequential blind and informed evaluation conditions. Four formulations containing 0%, 0.08%, 0.12%, and 0.16% xanthan gum were evaluated by 131 panelists using 30 mL tasting samples. Linear Mixed Models (LMMs) were applied to account for repeated measurements and inter-individual variability. Increasing viscosity systematically increased expected fullness while reducing overall liking and prospective hypothetical consumption volume. Panelists showed higher acceptance and greater prospective hypothetical consumption volume during the informed evaluation condition, although these differences were interpreted cautiously because the evaluation conditions were administered sequentially. Nevertheless, higher viscosity remained associated with lower hedonic ratings, indicating that oral texture continued to play a dominant role in product acceptance regardless of information disclosure. Taste liking was the strongest predictor of overall acceptance, whereas perceived viscosity was associated with lower prospective hypothetical consumption volume, particularly at higher xanthan gum concentrations. These findings highlight the importance of immediate sensory attributes, particularly taste and viscosity-related expected fullness, in shaping panelists’ acceptance and prospective hypothetical consumption volume. Differences between the sequential blind and informed evaluations, however, were interpreted cautiously because they may also reflect session-order and repeated-exposure effects.

## 1. Introduction

Dietary protein is essential for human health, contributing to muscle protein synthesis, metabolic regulation, and the maintenance of body composition [1,2,3]. Among dietary protein sources, whey protein has become a dominant ingredient in functional beverages because of its high solubility, complete amino acid profile, and superior bioavailability compared with many plant-based and casein proteins [4,5]. Despite these nutritional advantages, developing consumer-acceptable high-protein beverages remains challenging. Whey-based beverages often exhibit undesirable sensory characteristics, including a thin body, dry mouthfeel, and reduced overall palatability [6,7], which hydrocolloids like xanthan gum are commonly used to correct by enhancing rheology, thickness, and smoothness [8,9,10]. These physical modifications influence how consumers perceive beverages during oral processing. Higher beverage viscosity has frequently been associated with stronger expectations of fullness during consumption, although sensory evaluation primarily captures consumers’ immediate perceptions and expected fullness rather than physiological satiety [11,12,13]. Distinguishing these concepts is important for accurately interpreting consumer responses to viscosity modification in sensory studies. Even with these textural adjustments, a critical industry trade-off remains. While increased viscosity elevates immediate expected fullness [11,12], it simultaneously risks triggering sensory aversion. When thickness exceeds a panelist’s hedonic threshold, the texture is frequently perceived as cloying or unpalatable, leading to a sharp decline in palatability regardless of nutritional value. Identifying whether cognitive framing can mitigate this reduction in sensory liking is vital to maintaining product acceptability.

To address this sensory trade-off, sensory researchers increasingly examine extrinsic cognitive cues. Consumer acceptability is not governed solely by intrinsic physical attributes (e.g., rheology), but is strongly modulated by extrinsic information available before consumption, including nutritional claims and ingredient information [14,15,16]. Such information can shape perceived healthfulness and product expectations, thereby influencing hedonic evaluation even when the physical characteristics of the product remain unchanged. Consistent with this, previous studies have reported differences in consumer responses between blind and informed evaluations after nutritional information is disclosed [17,18,19,20]. Nevertheless, when blind and informed conditions are administered sequentially rather than counterbalanced, differences between evaluation conditions may reflect not only product information but also session-order effects, repeated sensory exposure, and increasing familiarity with the evaluation procedure. These methodological considerations should therefore be acknowledged when interpreting differences between evaluation conditions.

Although previous studies have investigated the effects of viscosity on sensory perception, expected fullness, and consumer acceptance, few have simultaneously examined sensory intensity, expected fullness, hedonic evaluation, and prospective hypothetical consumption volume of whey-based high-protein beverages under sequential blind and informed evaluation conditions. Furthermore, the relationships among these responses have rarely been evaluated simultaneously while accounting for repeated measurements and inter-individual variability among panelists using Linear Mixed Models.

Therefore, this study aimed to evaluate the effects of xanthan gum concentration on sensory intensity (viscosity, sweetness, and expected fullness), hedonic evaluation, and prospective hypothetical consumption volume of whey-based high-protein beverages under sequential blind and informed evaluation conditions. Linear Mixed Models were applied to account for repeated measurements and panelist-specific variability while examining the relationships among sensory perceptions and hedonic responses. By adopting a more cautious interpretation of inter-condition differences, this study provides further insight into how viscosity modification influences panelists’ sensory responses and acceptance of whey-based high-protein beverages under realistic sensory evaluation settings.

## 2. Materials and Methods

### 2.1. Physical Analysis

Physical analysis in the form of viscosity was conducted using a Brookfield DV2T series viscometer (AMETEK Broookfield, Middleboro, MA, USA) equipped with an LV-2 spindle. Measurements were performed at 24 ± 0.5 °C with a rotational speed of 60 rpm. The torque for all measurements was maintained within the recommended operating range of 10–100%. The spindle was dipped into the beaker containing a 100 mL sample up to the limit mark and the result appears on the viscometer screen in centiPoise (cP) units after stabilizing. Each formulation was measured in duplicate, and the average apparent viscosity was used for subsequent analysis. The high-protein beverage products were prepared at the Process Engineering Laboratory and the Food and Nutrition Laboratory, Faculty of Agricultural Technology, Universitas Gadjah Mada (UGM).

### 2.2. Sensory Analysis

This study used a dataset from sensory evaluation conducted in the Product Development and Sensory Evaluation Laboratory, Faculty of Agricultural Technology, UGM, Special Region of Yogyakarta, Indonesia, involving 131 untrained healthy adult volunteers (age 18–27 years; 30 males, 101 females) in 2023. A total of 144 untrained panelists were initially recruited through an open recruitment system on the first day of the sensory testing (Blind Condition). However, only 131 panelists attended the second day of sensory evaluation (Informed Condition). Following data screening and completeness assessment, data from 131 panelists with complete responses were included in the final statistical analysis. Panelists were recruited via digital platforms and provided written informed consent prior to the study. All panelists were screened to ensure compliance with the inclusion and exclusion criteria established in this study. The inclusion criteria were healthy individuals aged 18–40 years, willing to participate in the sensory evaluation procedures, not allergic to milk and dairy products, and residing in the Special Region of Yogyakarta. The exclusion criteria included individuals with impaired olfactory or gustatory function, lactose allergy, and those following a healthy lifestyle program. Panelists self-reported that they had no known taste or smell disorders at the time of the sensory evaluation and previous consumption of high-protein beverages was not used as an inclusion criterion. Panelists were recruited through voluntary convenience sampling and the screening was conducted through an online Google Form. Each participant received the same compensation after completing the evaluation, regardless of their responses.

The study protocol was approved by the Medical and Health Research Ethics Committee (MHREC), Faculty of Medicine, Public Health, and Nursing, Universitas Gadjah Mada—Dr. Sardjito General Hospital (Reference No. KE/FK/1578/EC/2023). To systematically isolate the cognitive expectations generated by labeling information from the physical sensory properties of the beverages, a deceptive experimental design incorporating manipulated nutritional claims was employed. Formal post-test debriefing was omitted following individual sensory sessions to preserve data validity and prevent information leakage among prospective participants within the shared community setting. Panelists completed the evaluations across two consecutive days, with the blind condition administered on Day 1 followed by the informed condition on Day 2. The blind evaluation was intentionally conducted first because nutritional information, once disclosed, cannot be unlearned or withdrawn without compromising baseline sensory responses; conversely, administering the informed condition first would have risked residual product familiarity or expectation carrying over into the subsequent blind session, compromising its validity. This sequencing is consistent with prior sensory research employing comparable blind-then-informed designs [17]. This fixed sequential order therefore ensured that participants remained completely naive to the nutritional framing during the initial assessment, thereby preventing cognitive contamination of sensory expectations. Each session involved evaluation of all four beverage formulations, and the two sessions were separated by a 24-h interval, both of which were intended to minimize sensory fatigue and confusion across the multi-sample protocol. Nevertheless, this fixed sequential design means that the observed condition effect is inherently confounded with repeated exposure, familiarity, memory, session, and day effects, and cannot be attributed to information disclosure alone. This confound is addressed explicitly in the interpretation of condition-related findings throughout the Results and Discussion, and is further discussed as a primary limitation of this study. Four high-protein beverage samples were formulated using Whey Protein Isolate 90 (Milk Specialties Global, Eden Prairie, MN, USA), skim milk powder, sugar, and water. Viscosity was manipulated by varying the concentration of xanthan gum (XG) at four levels: 0% (214), 0.08% (802), 0.12% (162), and 0.16% (785). The formula used to make high-protein drinks is described in Table 1.

The formulation employed a Completely Randomized Design with one factor, namely xanthan gum concentration. Four formulations, including one control, were prepared for sensory evaluation. The xanthan gum concentrations were selected based on concentrations commonly reported for beverage applications in the literature (0.001–0.15%) [9], with an additional 0.16% formulation included to extend the viscosity range evaluated. This range was further informed by a preliminary market survey of commercially available high-protein beverages conducted prior to formulation. Although the 0.16% concentration slightly exceeded the range reported in [9], no maximum limit for xanthan gum use in beverages is specified under the Indonesian Good Processed Food Manufacturing Practices (CPPB). XG was chosen due to its high thickening efficiency at low levels [9,21], and its high stability under acidic conditions and temperature variations, as well as its ability to dissolve readily in cold water, which is appropriate for whey protein beverages that are prepared without heat treatment to avoid protein denaturation [22,23].

The preparation process of the high-protein beverages with xanthan gum addition included ingredient weighing, dry mixing, sieving, mixing, and foam removal. All dry ingredients were thoroughly blended and passed through a 60-mesh sieve before beverage preparation. The beverage powder (30 g) was reconstituted in 200 mL of water at 25 °C and mixed using a high-speed mixer at 12,000 rpm for 10 s. After standing for 10 s, the surface foam was removed using a spoon. The prepared beverages were then stored under refrigerated conditions until sensory evaluation. Information intervention was provided to the panelists on the second day testing, immediately before the evaluation in the sensory booths along with testing instructions delivered by the panel leader. During the Informed session, panelists were provided with nutritional labels displaying specific protein claims corresponding to each sample (Table 2). To evaluate the interaction between physical properties and displayed information, the displayed protein claim—expressed as a percentage of the Nutritional Label Reference (% NLR)—was systematically varied alongside xanthan gum concentration, ranging from 5% NLR (0% XG) to 14% NLR (0.16% XG). It is important to emphasize that these figures represent experimental labeled information rather than actual chemical protein content, which remained identical across all formulations. Because the claim level co-varied with sample viscosity, the resulting differences between testing conditions reflect a combined effect of information presence, claim level, and viscosity expectations. The protein content of all beverage formulations was chemically determined using the micro-Kjeldahl method. Based on the Indonesian Food and Drug Authority (BPOM) Regulation No. 13 of 2016, all formulations met the criteria for high-protein products. The analytical results are provided in Supplementary Table S1.

Prior to the beginning of the sensory sessions, a standardized briefing was conducted to instruct panelists on the evaluation procedures and the digital questionnaire format, ensuring they were fully calibrated on how to complete the tasks. Sample preparation was assisted by facilitators who were also UGM students. The testing instructions and information sheets were additionally displayed in each booth. Panelists received a consistent sample volume of 30 mL served slightly chilled at approximately 10 °C in standardized, clear shot glasses. To eliminate expectation bias, each sample was labeled with a unique, three-digit random code (214, 802, 162, and 785). The same three-digit codes were used for all participants to maintain identical treatment conditions across all sensory comparisons. Because multiple panelists were evaluated concurrently in separate booths during each session, each panelist’s tray was individually prepared and verified by trained staff immediately prior to their session to prevent cross-panelist sample mix-up despite the shared coding scheme. However, the presentation order of the samples was fully counterbalanced across all participants using a systematic balanced presentation design encompassing all 24 possible permutations of the four-sample serving sequence, with panelists assigned to each permutation according to a fixed serving worksheet (Supplementary Table S2), to systematically control for potential first-order carryover and positional contrast effects. Each panelist was provided with an individual evaluation tray containing a sensory kit: room-temperature mineral water, a rinsing cup, a spoon, and laboratory tissues. Sensory evaluations were conducted in individual booths under controlled lighting conditions. Panelists evaluated the samples from left to right according to the assigned serving order, using mineral water and a clean spoon between consecutive samples to cleanse the palate and minimize sensory fatigue and cross-sample carryover. Ratings were recorded via digital forms (Google Forms). The evaluation required panelists to rate three primary dimensions of the product. First, perceived viscosity, sweetness, and expected fullness were evaluated using a 9-point structured intensity scale (1–9), anchored only at the endpoints (“low” to “high”), with no intermediate numerical descriptors, with higher scores indicating greater perceived intensity. Expected fullness was assessed following evaluation of a 30 mL sample, using the question ‘How full/satiated would you feel if you consumed 100 mL of this beverage?’ (1–9 scale). As panelists evaluated only a fraction (30 mL) of the referenced portion (100 mL), this measure reflects a forward-looking, cognitive projection of anticipated fullness rather than a physiological outcome or a concurrent oral sensation, consistent with the ‘expected fullness’ construct described by Brunstrom and Rogers [24] and methodologically grounded in the expected satiety paradigm of Brunstrom, Shakeshaft, and Scott-Samuel [25]. Second, hedonic ratings for overall liking and sub-attributes (taste, texture, and viscosity) were recorded using a 1–9 hedonic scale, ranging from “dislike extremely” (1) to “like extremely” (9). To quantify prospective consumption capacity, participants were asked to estimate the precise volume of each beverage sample they anticipated they could comfortably consume in a single sitting. The evaluation utilized a discrete scale with five level response in 50-mL steps: 50, 100, 150, 200, and 250 mL. The sensory evaluation sessions were conducted within two days and divided into 10 sessions each day, each session involving approximately 10–17 panelists.

### 2.3. Statistical Analysis

Physical test results were analyzed statistically using parametric methods, namely one-way ANOVA, followed by Tukey’s post-hoc test where significant differences were found. All analyses were performed using SPSS (Version 25.0; IBM Corp., Armonk, NY, USA) for Windows, with a significance level of 0.05. Sensory evaluation data, by contrast, were obtained from repeated assessments across sessions and therefore exhibit inherent hierarchical clustering, with multiple measurements nested within individual panelists [26]. Although ANOVA is commonly used for such data [27], inter-panelist and inter-session variation often violates the assumption of independence required by classical ANOVA, limiting the accuracy of treatment effect estimates, particularly in unbalanced designs or when data are missing [28]. To account for this correlated structure, Linear Mixed Models (LMMs) were utilized by specifying panelist identifiers (panelist_id) as random effects, accommodating repeated-measures correlation and inter-individual variability [26,29,30].

Analysis was performed using Stata (Version 19.0; StataCorp LLC, College Station, TX, USA). Model parameters were estimated via the Restricted Maximum Likelihood (REML) approach. Preliminary diagnostic checks were conducted using Ordinary Least Squares (OLS) regressions to evaluate potential multicollinearity. Variance Inflation Factors (VIFs) and Spearman rank correlation coefficients were monitored across all key predictors, with a VIF threshold of <5 indicating acceptable levels of collinearity [31]. Predictor variables were mean-centered prior to model estimation to enhance interpretability and minimize potential multicollinearity.

To evaluate the sensory profile and hedonic responses, four primary analytical frameworks were constructed using LMMs. First, to assess the impact of xanthan gum (XG) concentration and testing condition on sensory and hedonic attributes (Model 1), LMMs were specified for seven dependent variables: perceived viscosity, sweetness, expected fullness intensity, texture liking, taste liking, viscosity liking, and overall liking. These models evaluated continuous linear and quadratic terms of XG concentration, experimental condition (Blind vs. Informed), and their interactions, specifying panelists as random intercepts and random slopes for the linear XG term under an independent covariance structure. To control for Type I error across the seven parallel outcomes, Benjamini–Hochberg False Discovery Rate (FDR) corrections were applied [32]. Adjusted q-value was derived from the joint Wald test of the XG linear and quadratic terms evaluated at the baseline reference (Blind condition).

Second, to isolate sensory drivers independent of physical formulation (Model 2), overall liking was modeled against mean-centered intensity scores (viscosity, sweetness, expected fullness) and their interactions with condition, treating panelists as random intercepts. Quadratic terms were incorporated for texture and taste liking to detect non-linear penalties. Where significant curvature was identified, exact inflection points (vertices) were derived via the Delta method (nlcom command) to distinguish between optimal sensory boundaries and continuous declines within the observed range [33]. Third, overall liking was regressed against individual liking components (texture, taste, and viscosity liking) and their interactions with condition to determine the primary hedonic predictor of beverage acceptability (Model 3).

Finally, to explore the mechanism regulating consumption behavior, a sequential covariate adjustment protocol was applied to the anticipated consumption volume with an unadjusted baseline model evaluating linear and quadratic XG effects and condition interactions (Model 4U), a direct-effect model adjusted for expected fullness and sweetness (Model 4A), and a viscosity-adjusted model further accounting for perceived viscosity (Model 4B) to evaluate whether consumption boundaries were primarily driven by oral thickness constraints or expected fullness. As hypothetical consumption volume was recorded across discrete increments (50–250 mL), a mixed-effects ordered logistic regression (meologit) with panelist random intercepts was fitted as a sensitivity check. Structural consistency between continuous LMMs and ordinal logistic models validated the stability of statistical inferences. Residual diagnostics, including standardized residual plots and Quantile–Quantile plots, were visually inspected across all structural equations to confirm normality and homoscedasticity assumptions.

## 3. Results

### 3.1. Physical Analysis

**Figure 1:**
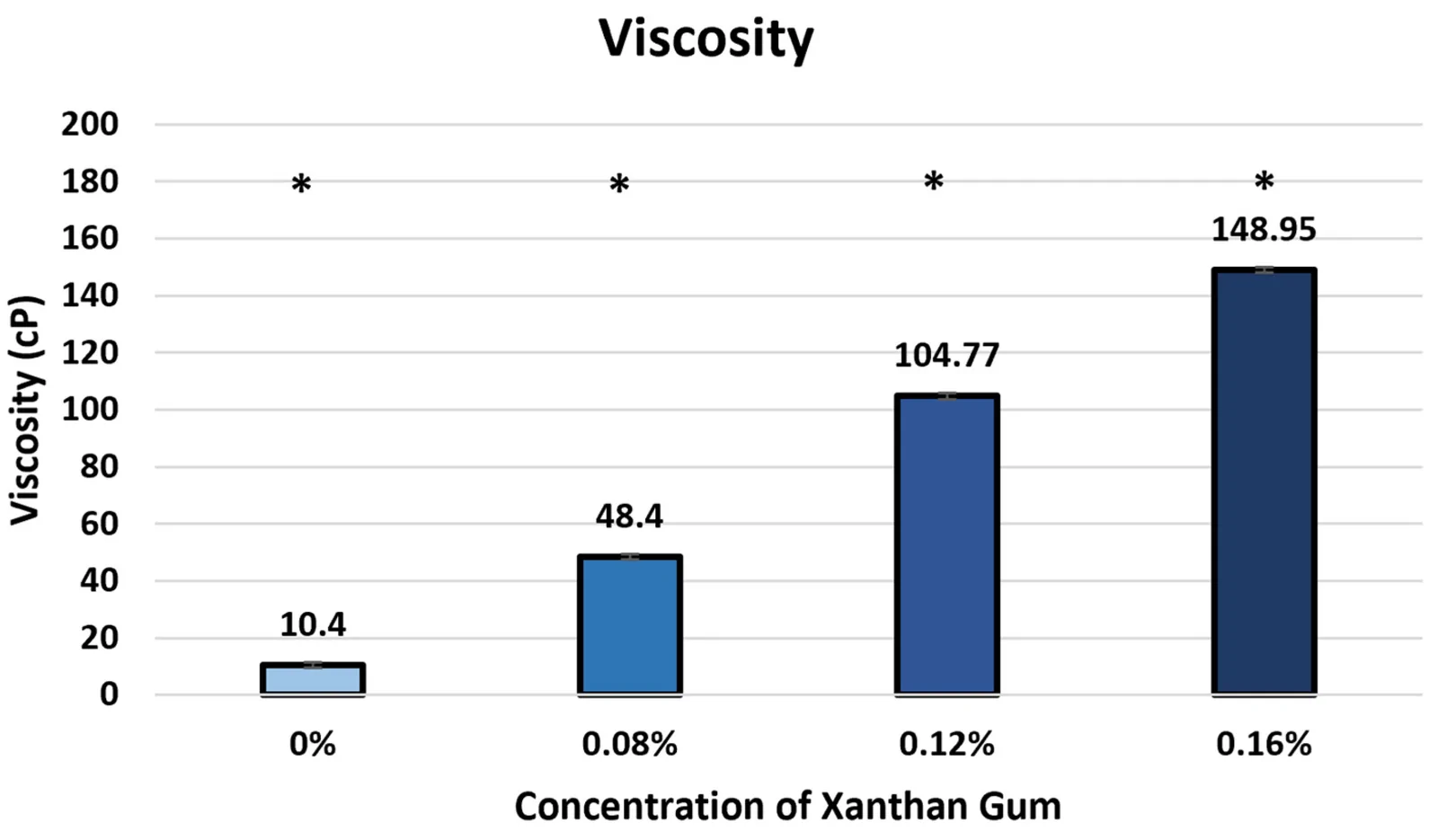
The result of viscosity for the four samples. The error bars show a 95% confidence interval. Asterisks show the significant effect *p* < 0.05.

From the analysis results, it can be seen that the addition of XG to whey protein beverage has a significant effect on its viscosity (*p* < 0.05). Figure 1 shows that the viscosity of high-protein beverages increased during the addition of XG concentration. The viscosity of high-protein beverages ranged between 10.40 and 148.95 cP. The sample without XG had the lowest viscosity, while the sample with 0.16% XG had the highest viscosity.

### 3.2. Sensory Analysis

Preliminary analysis using Spearman’s correlation indicated a moderate-to-strong association between expected fullness intensity and viscosity score (ρ = 0.65), whereas correlations involving sweetness were weak. Despite the observed correlation between expected fullness and viscosity, all predictors showed VIF values below 5, indicating the absence of severe multicollinearity. Accordingly, both variables were retained in the final Linear Mixed Models (LMMs) to capture their distinct contributions to the expected fullness mechanism.

The descriptive sensory intensities, hedonic scores, and prospective consumption volumes across the four empirical xanthan gum (XG) concentrations under both experimental conditions are summarized in Table 3. Across all samples, increasing xanthan gum concentration produced a clear monotonic increase in perceived viscosity ratings, rising from the lowest concentration baseline to the highest formulation. Concurrently, expected fullness intensity demonstrated a parallel upward trend across the concentration gradient, reflecting the immediate physical impact of hydrocolloid-induced fluid resistance during oral processing. In contrast, sweetness intensity scores exhibited a slight downward trend as XG concentration increased, suggesting a potential sensory suppression effect caused by the increased biopolymer matrix density. Regarding hedonic responses, overall liking and texture acceptance scores peaked at lower-to-intermediate XG concentrations, whereas higher concentrations received lower hedonic ratings.

To further evaluate the main and interaction effects of XG concentration and experimental condition on sensory profiles, Linear Mixed Models (LMMs) were estimated (Table 4). For viscosity, panelists’ perceived intensity showed a highly significant linear increase (β = 0.172, 95% CI [0.149, 0.195], *p* < 0.001) accompanied by a slight negative quadratic effect (β = −0.0066, 95% CI [−0.011, −0.002], *p* < 0.01). The Informed condition showed a lower baseline intercept (β = −0.403, 95% CI [−0.679, −0.127], *p* < 0.01), with a positive Condition × XG interaction (β = 0.037, 95% CI [0.004, 0.070], *p* < 0.05); as detailed in the Discussion, this condition effect is confounded with session order and repeated exposure and is interpreted accordingly throughout. Perceived sweetness decreased significantly with increasing XG concentration (β = −0.030, 95% CI [−0.050, −0.010], *p* < 0.01). Expected fullness intensity showed a strong positive linear association with XG concentration (β = 0.118, 95% CI [0.096, 0.140], *p* < 0.001), consistent with greater perceived viscosity being linked to higher anticipated fullness. Across hedonic attributes, all measures showed a significant negative linear association with XG concentration (e.g., Overall Liking: β = −0.051, 95% CI [−0.074, −0.028], *p* < 0.001). The informed condition was associated with significantly higher scores across all hedonic metrics (Texture Liking: β = 0.495, 95% CI [0.238, 0.752], *p* < 0.001; Overall Liking: β = 0.427, 95% CI [0.208, 0.647], *p* < 0.001), and the Condition × XG interaction was positive and significant for Viscosity Liking (β = 0.042, 95% CI [0.011, 0.073], *p* < 0.01) and Overall Liking (β = 0.030, 95% CI [0.003, 0.057], *p* < 0.05).

Associations between intensity and hedonic attributes are summarized in Table 5. Increases in sweetness intensity (β = −0.160, 95% CI [−0.248, −0.072], *p* < 0.001) and expected fullness intensity (β = −0.146, 95% CI [−0.225, −0.068], *p* < 0.001) showed the sharpest negative effects on overall liking, followed by viscosity intensity (β = −0.113, 95% CI [−0.183, −0.043], *p* = 0.002). These data indicate that formulations producing high expected fullness and viscosity were perceived as sensory impairments, resulting in lower overall liking scores.

Furthermore, when controlling for specific attribute hedonic scores (Texture, Taste, and Viscosity Liking) in Table 6, the main effect of Condition on Overall Liking became non-significant (β = −0.056, 95% CI [−0.128, 0.015], *p* = 0.120), and all interaction terms were also non-significant (*p* > 0.05). To distinguish the total effect of experimental condition from covariate-adjusted effects accounting for potential mediating pathways, condition effects on overall liking were compared across three model specifications with increasing levels of adjustment. In the unadjusted model (Table 4), controlling only for XG concentration as the design factor, the informed condition showed a significant total effect on overall liking (β = 0.427, 95% CI [0.208, 0.647], *p* < 0.001). After adjusting for sensory intensity covariates (viscosity, sweetness, and expected fullness intensity; Table 5), this effect attenuated substantially but remained significant (β = 0.185, 95% CI [0.034, 0.336], *p* = 0.017). After further adjusting for attribute-level hedonic scores (texture, taste, and viscosity liking; Table 6), the condition effect became non-significant (β = −0.056, 95% CI [−0.128, 0.015], *p* = 0.120). This pattern of progressive attenuation across increasingly adjusted specifications is presented in detail in the Discussion.

Finally, sequential adjustment modelling was performed to examine how xanthan gum (XG) concentration and experimental condition relate to hypothetical consumption volume in mL, and to evaluate the potential mediating roles of expected fullness, sweetness, and viscosity intensity (Table 7). In the unadjusted model (Model 4U), increasing XG concentration was associated with significantly lower hypothetical consumption volume (β = −3.716, 95% CI [−4.41, −3.03], *p* < 0.001), while volume estimates were significantly higher in the Informed condition (β = 20.77, 95% CI [13.54, 28.00], *p* < 0.001). After adjusting for expected fullness and sweetness intensity (Model 4A), the negative association between XG and hypothetical consumption volume was attenuated (β = −1.745, 95% CI [−2.40, −1.09], *p* < 0.001), with expected fullness intensity showing a strong independent negative association with volume estimates (β = −17.18, 95% CI [−19.22, −15.14], *p* < 0.001). When viscosity intensity was additionally included (Model 4B), it emerged as a further independent negative predictor (β = −7.02, 95% CI [−9.02, −5.02], *p* < 0.001), and the XG coefficient attenuated further (β = −1.065, 95% CI [−1.73, −0.41], *p* < 0.01). These attenuation patterns are examined further in Section 4.

To assess the robustness of the continuous linear mixed model (Model 4U), a random-intercept ordered logistic regression was conducted using hypothetical intake volume as a five-level ordered outcome (Supplementary Table S5). The ordered logistic model produced results consistent with the primary LMM. The linear effect of xanthan gum remained significantly negative (β = −0.191, *p* < 0.001), the quadratic term remained significantly positive (β = 0.0073, *p* = 0.017), and the Informed condition retained a significant positive association (β = 1.012, *p* < 0.001).

**Figure 2:**
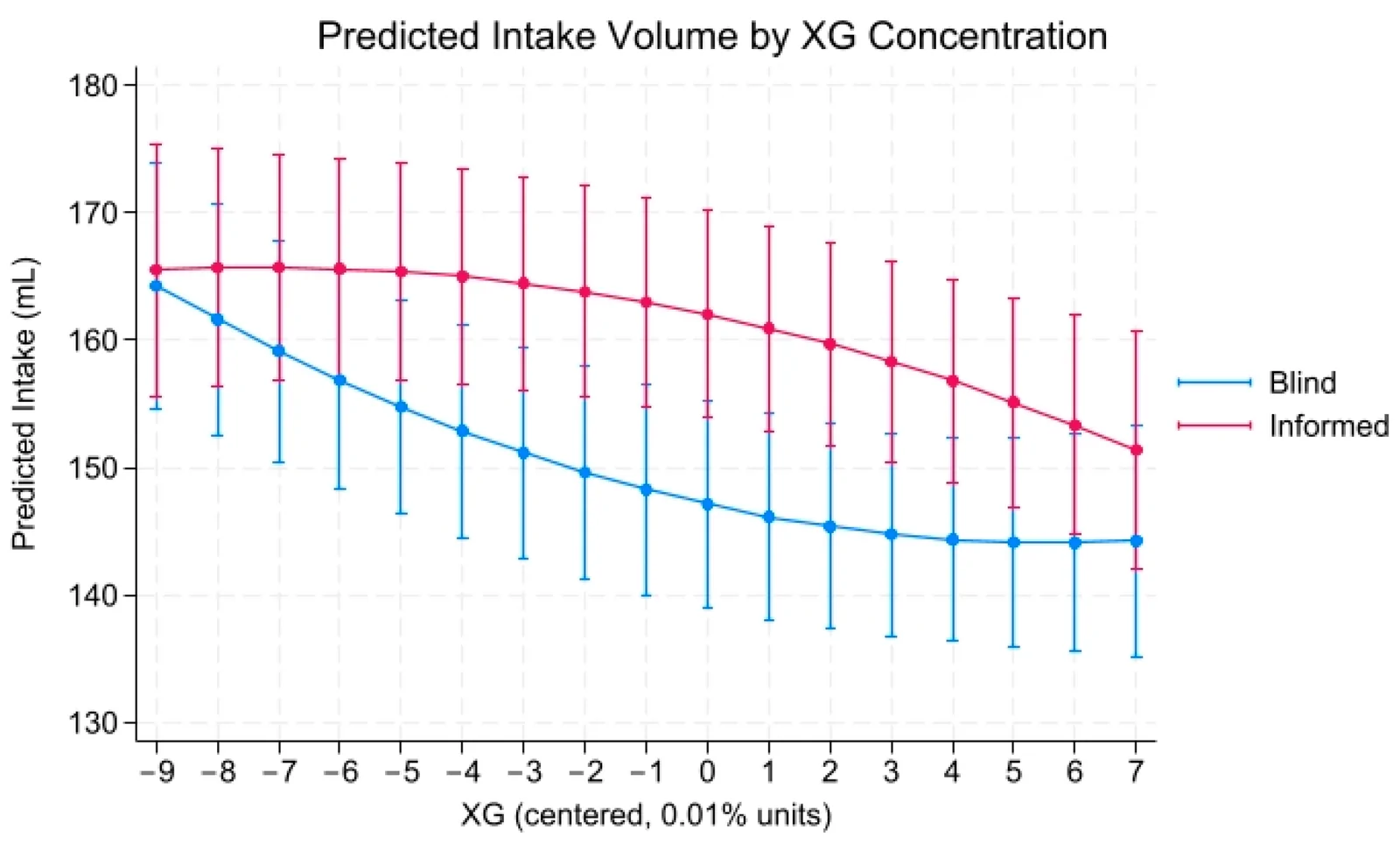
Model-predicted intake volume (mL) as a function of xanthan gum (XG) concentration (centered, 0.01% units) and experimental condition (Blind vs. Informed; N = 131), based on Model 4U (Table 7). Error bars represent 95% confidence intervals.

The adjusted behavioral trajectory was then visualized by plotting the predictive margins of the estimated hypothetical intake volume—derived from Model 4A—against the range of centered xanthan gum concentrations (Figure 2). Within the utilized formulation scale of −9 to 7, the estimated consumption volume dropped from approximately 164–165 mL at the lowest XG concentration to roughly 144 mL for the Blind group and 151 mL for the Informed group at the highest concentration. This visual trend confirms the significant negative linear impact of XG paired with the quadratic plateau effect on intake capacity found in the LMM analysis.

## 4. Discussion

Consumption of high-protein beverages is primarily driven by perceived ease and convenience [34]. Our findings indicate that variations in sample viscosity significantly influenced panelists’ preferences, as texture and consistency inherently modulate perceived flavor profiles [35]. High-protein beverages varied in viscosity following the xanthan gum (XG) concentration used in each sample; this increase in viscosity is attributed to the ability of XG to absorb water, leading to swelling and hydrogel formation in aqueous media [35,36]. The use of xanthan gum as a thickening agent effectively increased viscosity even at low concentrations, consistent with a positive correlation between XG addition and increased viscosity reported elsewhere [37,38,39,40], and with panelists’ evaluations of viscosity intensity in this study. Although higher viscosity is known to be associated with greater expected fullness compared to low-viscosity alternatives [36,41], as reflected in the intensity attributes shown in Table 3, it also poses challenges to overall acceptability. Panelists consistently preferred samples with little or no added thickener, likely because the resulting mouthfeel was perceived as unfamiliar or deviated from conventional milk-based beverages. Previous research revealed that adding xanthan gum to apple juice caused a decrease in panelists’ preference for mouthfeel. The decreased preference for the mouthfeel of apple juice is caused by the level of viscosity that creates a “slimy” impression and the mouthfeel produced from juice with xanthan gum is not like apple juice in general [42]. This decrease in preference may also reflect the product’s viscosity exceeding the maximum acceptable limit of 31.6 cP reported in prior work [43], beyond which panelists find thickened beverages difficult to accept. Overall, panelists preferred high-protein drink samples without added xanthan gum, with viscosity resembling conventional milk, over higher-viscosity formulations.

A central finding of this study is the strong linear relationship between XG concentration and expected fullness. Assessed immediately after tasting 30 mL samples, higher biopolymer density systematically increased oral perceptions of thickness, smoothness/slipperiness, and fluid resistance, consistent with prior studies showing that hydrocolloid-thickened matrices alter dynamic texture perception during brief oral contact [44,45,46]. Because testing involved brief exposure to a small sample volume relative to the projected 100 mL portion referenced in the expected fullness question, these evaluations are interpreted as a forward-looking, texture-driven anticipation of fullness rather than systemic, postprandial physiological satiety or metabolic appetite regulation [36,47,48]. Regarding overall acceptance, estimations in Table 5 revealed that sweetness intensity, viscosity intensity, and expected fullness intensity were all negatively associated with overall liking. This decline can be attributed to sensory imbalance: sweetness typically triggers a direct hedonic response via the brain’s reward pathways [49], whereas excessive viscosity produce a dominant sensation of viscosity that overshadows other pleasant sensory attributes [50]. A quadratic response surface analysis (Supplementary Table S6) further clarifies the nature of this sensory aversion. Rather than displaying an inverted U-shaped curve or a sensory “bliss point,” the relationship between sensory intensity and liking is characterized by a monotonic decline that accelerates at higher intensity levels. Because the mathematical peak point sits at the lower end of the observed centered range, no optimal sensory threshold exists within the tested formulations, indicating that increases in perceived thickness or sweetness beyond baseline levels trigger a progressively faster decline in liking. This is notable given that satiety-related constructs are often reported in the literature primarily as post-consumption outcomes rather than direct, unmediated drivers of liking [51], underscoring that the expected fullness measured here reflects an anticipatory, pre-consumption judgment distinct from that literature.

Panelists generally assigned higher hedonic scores and reported greater hypothetical consumption volume estimates during the Informed condition than during the Blind condition. This pattern aligns with prior literature demonstrating that external product information and protein claims can modulate consumer perception, emotion, and overall sensory liking [17,20], often inducing a positive halo effect that enhances the overall sensory experience [52]. However, because the Blind and Informed conditions were administered sequentially rather than counterbalanced, these observed differences cannot be attributed solely to the provision of product information. Previous sensory research has demonstrated that carry-over, question-order, and presentation-order effects can independently influence hedonic and acceptability ratings, highlighting the importance of carefully interpreting differences observed across sequential sensory evaluations [53,54,55]. Consequently, the elevated scores observed in the Informed condition should be interpreted as reflecting differences between sequential evaluation conditions, in which product information disclosure was inherently confounded with repeated exposure, familiarity, memory effects, and session-order dynamics, rather than as evidence of an isolated information effect.

Despite these session dynamics, another notable pattern in this study was the apparent primacy of sensory experience over provided product information. Providing health-related information on food items has been shown to influence consumer preferences, including snack choices [56], and adequate protein intake is recognized as supporting overall health and contributing to the management of chronic metabolic conditions [57]. In the present study, despite panelists being informed that higher-viscosity samples corresponded to higher labeled protein content, findings from Table 6 revealed no significant interaction between the condition factor and sub-hedonic attributes (Texture, Taste, and Viscosity Liking) on overall liking (*p* > 0.05). Panelists’ liking scores steadily declined as viscosity increased, regardless of whether product information was disclosed. Notably, controlling for these specific attribute hedonic scores caused the main effect of Condition on overall liking to become non-significant (β = −0.056, 95% CI [−0.128, 0.015], *p* = 0.120), compared with the significant condition effect observed in the unadjusted model (Table 4; β = 0.427, *p* < 0.001). This pattern is consistent with the elevated baseline acceptance observed in the unadjusted model operating through, rather than independently of, attribute-level liking—suggesting that the apparent effect of session condition on overall liking is substantially mediated through changes in texture, taste, and viscosity perception, rather than acting as an independent driver. Formal mediation testing (e.g., indirect-effect estimation) was beyond the scope of the present analysis and is noted as a direction for future work. In other words, while product information or marketing may elevate baseline appeal, it does not appear to offset the negative impact of suboptimal sensory attributes such as excessive viscosity. Panelists remained consistent in their attribute-level evaluations regardless of disclosed information, indicating that immediate sensory gratification—particularly taste and mouthfeel balance—remained the primary determinant of product acceptance within this young adult panel. These results are consistent with previous work showing that sensory experience often outweighs informational cues in shaping consumer liking. Lee and Lee [58] reported minimal influence of oil-free product information on overall liking, and Meier-Dinkel et al. [59] similarly found that product label information did not substantially alter hedonic responses. Sub-attribute analysis further confirmed taste liking as the strongest predictor of overall liking, ahead of texture or viscosity, emphasizing that holistic consumer acceptance is driven more strongly by taste than by texture or viscosity alone [60]. This is consistent with evidence that consumers care about healthy eating in principle yet rarely translate nutrition-label information into actual consumption choices, with “wants” and perceived benefit exerting the greatest influence on snack food choice [61].

Beyond preference, hydrocolloid rheology and session dynamics functioned as key correlates of hypothetical consumption volume (Table 7). Across formulations, the Informed condition was associated with higher volume estimates than the Blind condition (Figure 2), though physical thickness exerted a consistent negative association, attenuating by approximately 53% after adjusting for expected fullness and sweetness intensity (Model 4A). This attenuation pattern is consistent with texture modification playing a role in shaping anticipated consumption capacity, potentially related to slower oral processing, as suggested elsewhere [41,62,63], although the current study design cannot directly confirm the mechanism, as it is based on self-reported estimates of hypothetical consumption volumes rather than measured intake. Because expected fullness was assessed after the hypothetical consumption volume decision within the experimental sequence, this attenuation is reported as a close statistical association rather than a strict causal mediation pathway. When perceived viscosity intensity was additionally included (Model 4B), it emerged as a further significant negative predictor, and the positive quadratic (plateau) term weakened substantially. Because perceived viscosity intensity was evaluated prior to the volume decision, this weakening of the plateau term is more plausibly interpreted as reflecting an early sensory cue that shapes expected fullness, rather than as a downstream consequence of fullness itself [12,47,64].

The robustness of these findings is further supported by the LMM framework, in which the Likelihood Ratio (LR) test (Prob ≥ chibar2 = 0.0000) confirmed the significance of panelist-level random effects [65]. Significant random intercepts and slopes indicate substantial inter individual variability in perceptions of taste and expected fullness [66]. Incorporating random slopes was therefore essential to accurately estimate fixed effects across a heterogeneous panel population [29,67,68]. This approach captures individual differences in sensory sensitivity and response patterns, reflecting inherent disagreement among panelists [69]. To ensure that the behavioral inferences were insensitive to the continuous approach to the consumption volume scale, a random-intercept ordered logistic regression analysis was run as a complementary robustness test (Supplementary Table S5). The resulting consistency between the ordered logistic model and the primary LMM indicates that the observed associations were robust across alternative statistical frameworks. This cross-model consistency demonstrates that the associations of viscosity and information disclosure with prospective consumption volume are structurally stable and reliable, rather than being dependent on the choice of statistical estimation method.

From an application perspective, increasing xanthan gum may serve as one approach for enhancing expected fullness in protein beverages formulations, although this must be balanced against its tendency to reduce short-term panelist liking. This liking penalty may be minimized by optimizing taste profiles rather than relying primarily on nutritional claims. While panelists in this study prioritized sensory attributes such as taste and mouthfeel over conceptual product information, appropriate labeling and transparent disclosure remain valuable tools for aligning product expectations with health-related perceptions. Industry developers are thus encouraged to design high-protein formulations that harmonize nutritional value with tailored sensory profiles, for example, familiar flavor variants such as chocolate, strawberry, or vanilla, to enhance overall acceptance without compromising structural mouthfeel characteristics. These findings suggest that, alongside marketing and labeling strategies, physical/rheological formulation design remains an important lever for shaping consumer expectations of fullness and anticipated consumption in functional protein beverage development.

### Study Limitations

Despite the robustness of the analytical approach, this study has several limitations.

**Fixed condition order.** The experimental design employed a fixed sequential order (Blind on Day 1, Informed on Day 2) without counterbalancing across participants. Consequently, differences between evaluation conditions are inherently confounded with session order, repeated exposure, familiarity, and memory effects, and should not be interpreted solely as the effect of product information.

**Deceptive information framing.** The Informed condition involved presenting participants with modified nutritional labeling to systematically manipulate perceived formulation information. While this is a common paradigm in expectation-based sensory research, it raises considerations regarding participant awareness and response authenticity. Participants were not debriefed regarding this manipulation following the study, which should be addressed in future protocols.

**Hypothetical consumption volume versus actual consumption.** Expected fullness and hypothetical consumption volume were assessed as short-term, self-reported perceptual judgments following a 30 mL tasting sample, rather than through actual food consumption, postprandial physiological response, or gastric emptying. Because these attributes were assessed within the same experimental framework as the outcome measures, observed relationships were interpreted as associative rather than strictly causal.

**Absence of physiological satiety data.** Panelists’ baseline hunger state and prior food intake were not standardized or recorded before testing. Consequently, findings were not interpreted as reflecting physiological satiety or actual intake regulation. Future studies may incorporate standardized pre-test fasting protocols and physiological satiety measures to strengthen future findings.

**Incomplete rheological characterization.** Rheological characterization was limited to single-point apparent viscosity measurements without full flow curves, shear-rate modeling, or comprehensive physicochemical characterization (e.g., pH, hydration duration, storage conditions).

**Participant demographics and sampling.** Participants were recruited through voluntary convenience sampling from a single geographical region (Special Region of Yogyakarta) and consisted predominantly of young female university students. Habitual consumption of high-protein beverages was not an inclusion criterion, and taste/smell disorders were screened by self-report rather than objective testing. Although sample presentation order was randomized and counterbalanced, identical sample codes were used across participants within each session, and minor response or presentation bias cannot be completely excluded. Compensation was provided at a flat rate to all participants upon completion, independent of their individual ratings, though the theoretical possibility of mild response or social desirability bias cannot be entirely ruled out. These findings should therefore not be generalized beyond populations with similar demographic characteristics.

Future studies should validate the present findings using mixed designs that manipulate information between independent groups while preserving within-subject sensitivity to viscosity, delayed post-study debriefing, standardized pre-test fasting protocols, comprehensive physiological and rheological characterization, alternative hydrocolloids, and a priori power calculations to support broader, demographically representative consumer panels across diverse age, gender, and geographic profiles evaluated under more naturalistic consumption conditions.

## 5. Conclusions

The present study used Linear Mixed Models (LMMs) to evaluate sensory responses while accounting for heterogeneity among panelists. The results indicate that the sensory evaluation of whey-based high-protein beverages is primarily driven by physical sensory attributes, whereas differences between the sequential Blind and Informed evaluation conditions were interpreted as reflecting a combination of information exposure, repeated tasting, and session-order effects and were substantially mediated through attribute-level hedonic responses rather than acting independently. Increasing xanthan gum concentration consistently increased expected fullness while reducing overall liking and hypothetical consumption volume, indicating that excessive viscosity remained a major sensory constraint despite the intervention of protein-related information. Taste and mouthfeel remained the strongest determinants of product acceptance, suggesting that optimizing sensory quality is likely to be more effective than relying solely on protein-related information when developing thickened high-protein beverages.

From a product development perspective, these findings provide practical guidance for the formulation of high-protein beverages. While communicating protein-related information may improve consumer expectations during product evaluation, achieving an appropriate balance between flavor and viscosity remains essential for maximizing consumer acceptance. Because this study was conducted under controlled laboratory conditions involving manipulated nutritional information and using a predominantly young university-based sample from a single geographical region, the findings reflect immediate perceptual and cognitive responses within this experimental context and should not be generalized to broader consumer populations or real-world consumption behavior without further validation. Future research should consider determining the consumer rejection threshold for beverage viscosity. Additionally, treatment levels should be based on actual viscosity rather than solely on the percentage of xanthan gum added.

## Figures and Tables

**Table 1:** High-protein beverage ingredient formulations.

Material		Amount (g)				Amount (%)		
	214	802	162	785	214	802	162	785
Whey Protein Isolate 90	30	30	30	30	12.00	11.99	11.99	11.98
Skim Milk Powder	15	15	15	15	6.00	6.00	5.99	5.99
Sugar	5	5	5	5	2.00	2.00	2.00	2.00
Water	200	200	200	200	80	79.94	79.9	79.87
Xanthan gum (XG)	0	0.2	0.3	0.4	0	0.08	0.12	0.16

**Table 2:** Labeled protein information presented to panelists during the informed session.

Sample	Labeled Protein Claim (% NLR)
214 (0% XG)	5% NLR
802 (0.08% XG)	8% NLR
162 (0.12% XG)	11% NLR (may qualify for a protein source claim under BPOM RI No. 9/2016)
785 (0.16% XG)	14% NLR (may qualify for a protein source claim under BPOM RI No. 9/2016)

NLR = Nutritional Label Reference. Values reflect the experimental protein information displayed to panelists, not actual analytical protein content (which was held constant across samples). Based on the Indonesian Food and Drug Authority (BPOM RI) Regulation No. 9 of 2016 regarding Nutritional Label Reference (*Acuan Label Gizi* or *ALG*), a liquid product can be claimed as a ‘Source of Protein’ if it contains at least 10% of the NLR per 100 mL (equivalent to 6 g/100 mL, based on a general NLR of 60 g protein). A ‘High Protein’ claim requires at least 17.5% of the NLR per 100 mL (10.5 g/100 mL).

**Table 3:** Descriptive statistics of sensory attributes and hedonic scores across XG concentrations and experimental conditions.

Attribute	Condition	Sample 214 (0.00% XG)	Sample 802 (0.08% XG)	Sample 162 (0.12% XG)	Sample 785 (0.16% XG)
Perceived intensity attributes (1–9)					
Viscosity	Blind	3.63 (1.73)	5.60 (1.87)	6.13 (2.03)	6.63 (1.70)
	Informed	3.20 (1.53)	5.15 (1.83)	5.88 (1.95)	6.66 (1.69)
Expected fullness	Blind	5.05 (1.76)	5.92 (1.73)	6.51 (1.77)	6.89 (1.57)
	Informed	4.79 (1.65)	5.77 (1.78)	6.29 (1.65)	6.78 (1.61)
Sweetness	Blind	6.59 (1.41)	6.72 (1.46)	6.52 (1.63)	6.26 (1.62)
	Informed	6.00 (1.65)	6.17 (1.50)	6.20 (1.49)	5.82 (1.62)
Hedonic attributes for liking (1–9)					
Texture	Blind	7.11 (1.81)	6.31 (1.97)	5.98 (2.29)	5.67 (2.17)
	Informed	6.98 (1.89)	6.78 (1.59)	6.51 (1.87)	6.16 (1.90)
Taste	Blind	6.47 (2.04)	6.25 (1.83)	5.98 (2.11)	5.95 (2.09)
	Informed	6.61 (1.96)	6.63 (1.73)	6.54 (1.84)	6.60 (1.72)
Viscosity	Blind	6.69 (1.90)	6.20 (1.98)	5.85 (2.19)	5.73 (2.08)
	Informed	6.59 (2.11)	6.38 (1.84)	6.44 (1.81)	6.26 (1.92)
Overall	Blind	6.85 (1.70)	6.33 (1.66)	5.95 (2.05)	6.01 (1.89)
	Informed	6.71 (1.86)	6.63 (1.57)	6.56 (1.71)	6.41 (1.69)
Hypothetical consumption volume (mL)					
Volume estimates	Blind	187.79 (55.15)	147.71 (58.46)	131.30 (61.50)	124.43 (61.20)
	Informed	195.80 (54.79)	169.47 (57.37)	148.47 (59.14)	135.11 (58.41)

Note. Values are presented as Mean (Standard Deviation).

**Table 4:** Linear mixed model estimates for perceived sensory attributes and hedonic evaluations across xanthan gum (XG) concentrations and testing conditions (Blind vs. Informed, N = 131).

Attribute	XG Linear (β)	XG Quadratic (β)	Informed Condition, (Ref: Blind)	Condition × XG	*q*-Value (Benjamini–Hochberg)
Perceived viscosity	0.172 (0.012) ***	−0.0066 (0.0022) **	−0.403 (0.141) **	0.037 (0.017) *	<0.001
Perceived sweetness	−0.030 (0.010) **	−0.0043 (0.0018) *	−0.457 (0.117) ***	0.011 (0.014)	0.008
Expected fullness	0.118 (0.011) ***	0.0002 (0.0018)	−0.182 (0.117)	0.007 (0.014)	<0.001
Texture liking	−0.087 (0.014) ***	0.0013 (0.0021)	0.495 (0.131) ***	0.029 (0.016)	<0.001
Taste liking	−0.035 (0.012) **	0.0001 (0.0020)	0.435 (0.128) ***	0.032 (0.015) *	0.008
Viscosity liking	−0.060 (0.015) ***	0.0008 (0.0021)	0.327 (0.135) *	0.042 (0.016) **	<0.001
Overall liking	−0.051 (0.012) ***	0.0027 (0.0018)	0.427 (0.112) ***	0.030 (0.014) *	<0.001

Notes: β (SE); * *p* < 0.05, ** *p* < 0.01, *** *p* < 0.001. The 95% Confidence Intervals (CIs) are reported in the text (for primary effects) and Supplementary Table S3 (for all terms).

**Table 5:** Linear mixed model estimation of sensory intensity attributes (viscosity, sweetness, expected fullness) as drivers of overall liking, controlling for experimental condition (Blind vs. Informed; N = 131).

Predictor Variables	Coefficient (β)	SE	95% CI	*p*-Value
Fixed effects				
Viscosity intensity	−0.113	0.036	−0.183, −0.043	0.002
Expected fullness intensity	−0.146	0.040	−0.225, −0.068	<0.001
Sweetness intensity	−0.160	0.045	−0.248, −0.072	<0.001
Informed (condition)	0.185	0.077	0.034, 0.336	<0.017
Interactions				
Condition × Viscosity	−0.00003	0.047	−0.093, 0.092	0.995
Condition × Sweetness	0.038	0.052	−0.064, 0.140	0.471
Condition × Expected fullness	0.130	0.057	0.018, 0.241	0.022
Random effects				
Var(intercept)	1.636	0.227	1.246, 2.147	
Var(residual)	1.488	0.070	1.358, 1.632	

Note. Experimental condition treated as binary (reference: Blind).

**Table 6:** Covariate-adjusted linear mixed model for overall liking, controlling for attribute-level hedonic dimensions (texture, taste, and viscosity liking) and condition (Blind vs. Informed; N = 131).

Predictor Variable	Coefficient (β)	SE	95% CI	*p*-Value
Fixed effects				
Texture hedonic	0.299	0.020	0.259, 0.337	***
Taste hedonic	0.408	0.019	0.370, 0.445	***
Viscosity hedonic	0.233	0.019	0.195, 0.270	***
Condition (1)	−0.056	0.036	−0.128, 0.015	0.120
Interactions				
Condition—Texture hedonic	0.021	0.031	−0.040, 0.082	0.504
Condition—Taste hedonic	−0.016	0.028	−0.071, 0.039	0.569
Condition—Viscosity hedonic	0.034	0.029	−0.022, 0.091	0.234
Random effects (variance)	Estimate			
Var(intercept)	0.063	0.013	0.042, 0.095	—
Var(residual)	0.339	0.016	0.309, 0.372	—

Note. *** *p* < 0.001.

**Table 7:** Sequential linear mixed models evaluating XG concentration and condition (Blind vs. Informed; N = 131) as predictors of hypothetical consumption volume (mL; 5-level discrete scale, 50–250 mL), adjusting for expected fullness, sweetness, and viscosity intensity.

Term	Model 4U (Total)	Model 4A (+Exp. Fullness, Sweetness)	Model 4B (+Viscosity)
XG Linear	−3.716 (0.352) ***	−1.745 (0.335) ***	−1.065 (0.337) **
XG quadratic	0.146 (0.058) *	0.141 (0.048) **	0.093 (0.047) *
Informed (condition)	20.77 (3.69) ***	16.85 (3.10) ***	14.91 (2.98) ***
Interactions			
Condition × XG linear	−0.235 (0.445)	−0.313 (0.404)	0.041 (0.424)
Condition × XG quadratic	−0.182 (0.082) *	−0.191 (0.068) **	−0.164 (0.066) *
Expected fullness intensity	—	−17.18 (1.04) ***	−12.70 (1.18) ***
Perceived sweetness intensity	—	−1.83 (1.09)	−1.90 (1.05)
Perceived viscosity intensity	—	—	−7.02 (1.02) ***
Random effect	Estimate		
Var(intercept)	1875.1	1601.2	1696.4
Var(XG slope)	3.222	3.539	3.172
Var(residual)	1410.2	975.1	895.0
Log-likelihood	−5477.27	−5305.24	−5265.68

Notes. β (SE); * *p* < 0.05, ** *p* < 0.01, *** *p* < 0.001. The 95% CIs are in Supplementary Table S4.

## Data Availability

The original contributions presented in this study are included in the article/Supplementary Materials. Further inquiries can be directed to the corresponding authors.

## Supplementary Materials

The following supporting information can be downloaded at https://www.mdpi.com/article/10.3390/foods15162871/s1. Supplementary Table S1. Chemical analysis: Protein content and Nutrition Label References (NLR) adequacy per 100 grams. Supplementary Table S2. Sensory Evaluation Sample Set Presentation Design Worksheet. Supplementary Table S3. Full linear mixed model estimates (β, SE, 95% CI, and overall model q-value) for perceived sensory attributes and hedonic evaluations across xanthan gum concentrations and testing conditions (Blind vs. Informed; N = 131). Supplementary Table S4. Full model estimates (β, SE, 95% CI) for Models 4U, 4A, and 4B (Table 7). Supplementary Table S5. Ordinal-logit robustness check for intake, compared with the continuous LMM (Model 4U). Supplementary Table S6. Curvature of the sensory-to-liking relationships, with vertex location. Supplementary Table S7. Random effects variance components for Table 4.
