# Supplementary material for: Effects of Viscosity Modification on Sensory Perception, Hedonic Evaluation, and Expected Fullness of Whey-Based High-Protein Beverages Under Blind and Informed Conditions
Source: Foods. 2026 Aug 17;15(16):2871. doi: 10.3390/foods15162871 (PMC13512243; doi:10.3390/foods15162871)
Supplement: Supplementary file 1 [file foods-15-02871-s001.zip › foods-4432302-supplementary.pdf]

Supplementary Tables

Supplementary Table S1. Chemical analysis: Protein content and Nutrition Label References (NLR) adequacy per 100 grams

XG (%)	Protein Content (%)	Protein Content/100 gram	NLR adequacy(%)
0%	50.80 ± 1.699[a]	50.8	84.67
0,08%	49.65 ± 1.809[a]	49.65	82.75
0,12%	49.25 ± 2.895[a]	49.25	82.08
0,16%	48.84 ± 1.814[a]	48.84	81.40

Notes: Values with different superscript letters in each column indicate significant differences (at $\alpha = 0.05$). All four samples meet the requirements for the "high-protein" claim for powdered beverages, as they all have an NLR percentage greater than 35%.

Supplementary Table S2. Sensory Evaluation Sample Set Presentation Design Worksheet

Serving Worksheet					
Panelist					Sample Code (serving order: left to the right)
1	25	49	73	97	214, 802, 162, 785
2	26	50	74	98	214, 162, 802, 785
3	27	51	75	99	214, 802, 785, 162
4	28	52	76	100	214, 785, 802, 162
5	29	53	77	101	214, 162, 785, 802
6	30	54	78	102	214, 785, 162, 802
7	31	55	79	103	802, 214, 162, 785
8	32	56	80	104	802, 214, 785, 162
9	33	57	81	105	802, 162, 214, 785
10	34	58	82	106	802, 162, 785, 214
11	35	59	83	107	802, 785, 162, 214
12	36	60	84	108	802, 785, 214, 162
13	37	61	85	109	162, 214, 802, 785
14	38	62	86	110	162, 214, 785, 802
15	39	63	87	111	162, 802, 214, 785
16	40	64	88	112	162, 802, 785, 214
17	41	65	89	113	162, 785, 214, 802

18	42	66	90	114	162, 785, 802, 214
19	43	67	91	115	785, 214, 802, 162
20	44	68	92	116	785, 214, 162, 802
21	45	69	93	117	785, 802, 214, 162
22	46	70	94	118	785, 802, 162, 214
23	47	71	95	119	785, 162, 214, 802
24	48	72	96	120	785, 162, 802, 214

Supplementary Table S3. Full linear mixed model estimates (β, SE, 95% CI, and overall model q-value) for perceived sensory attributes and hedonic evaluations across xanthan gum concentrations and testing conditions (Blind vs. Informed; N = 131).

Attribute	Term	β (SE)	95% CI Lower	95% CI Upper	q-value
Perceived viscosity	XG linear	0.172 (0.012)***	0.149	0.195	<0.001
	XG quadratic	-0.0066 (0.0022)**	-0.011	-0.002	
	Informed (condition)	-0.403 (0.141)**	-0.679	-0.127	
	Condition × XG	0.037 (0.017)*	0.004	0.070	
Perceived sweetness	XG linear	-0.030 (0.010)**	-0.050	-0.010	0.008
	XG quadratic	-0.0043 (0.0018)*	-0.008	-0.001	
	Informed (condition)	-0.457 (0.117)***	-0.686	-0.228	
	Condition × XG	0.011 (0.014)	-0.016	0.038	
Expected fullness	XG linear	0.118 (0.011)***	0.096	0.140	<0.001
	XG quadratic	0.0002 (0.0018)	-0.003	0.004	
	Informed (condition)	-0.182 (0.117)	-0.411	0.047	
	Condition × XG	0.007 (0.017)	-0.026	0.040	
Texture liking	XG linear	-0.087 (0.014)***	-0.114	-0.060	<0.001
	XG quadratic	0.0013 (0.0021)	-0.003	0.005	
	Informed (condition)	0.495 (0.131)***	0.238	0.752	
	Condition × XG	0.029 (0.016)	-0.002	0.060	
Taste liking	XG linear	-0.035 (0.012)**	-0.058	-0.012	0.008
	XG quadratic	0.0001 (0.0020)	-0.004	0.004	
	Informed (condition)	0.435 (0.128)***	0.184	0.686	
	Condition × XG	0.032 (0.015)*	0.003	0.061	
Viscosity liking	XG linear	-0.060 (0.015)***	-0.089	-0.031	<0.001
	XG quadratic	0.0008 (0.0021)	-0.003	0.005	
	Informed (condition)	0.327 (0.135)*	0.062	0.592	
	Condition × XG	0.042 (0.016)**	0.011	0.073	
Overall liking	XG linear	-0.051 (0.012)***	-0.074	-0.028	<0.001
	XG quadratic	0.0027 (0.0018)	-0.001	0.006	
	Informed (condition)	0.427 (0.112)***	0.208	0.647	
	Condition × XG	0.030 (0.014)*	0.003	0.057	

Note. β (SE) and 95% CI for all fixed effects, including quadratic and interaction terms. Asterisks indicate significance based on adjusted p-values: *p<0.05, **p<0.01, ***p<0.001. The q-value (Benjamini-Hochberg corrected) reflects the overall model significance test for each attribute and is reported once per attribute.

Supplementary Table S4. Full model estimates (β, SE, and 95% CI) for Models 4U, 4A, and 4B (Table 7).

Model	Term	β (SE)	95% CI Lower	95% CI Upper
4U	XG linear	-3.716 (0.352)***	-4.41	-3.03
4U	XG quadratic	0.146 (0.058)*	0.03	0.26
4U	Informed (condition)	20.77 (3.69)***	13.54	28.00
4U	Condition × XG linear	-0.235 (0.445)	-1.11	0.64
4U	Condition × XG quadratic	-0.182 (0.082)*	-0.34	-0.02
4A	XG linear	-1.745 (0.335)***	-2.40	-1.09
4A	XG quadratic	0.141 (0.048)**	0.05	0.24
4A	Informed (condition)	16.85 (3.10)***	10.77	22.93
4A	Condition × XG linear	-0.313 (0.404)	-1.10	0.48
4A	Condition × XG quadratic	-0.191 (0.068)**	-0.32	-0.06
4A	Expected fullness intensity	-17.18 (1.04)***	-19.22	-15.14
4A	Perceived sweetness intensity	-1.83 (1.09)	-3.97	0.31
4B	XG linear	-1.065 (0.337)**	-1.73	-0.40
4B	XG quadratic	0.093 (0.047)*	0.00	0.19
4B	Informed (condition)	14.91 (2.98)***	9.07	20.75
4B	Condition × XG linear	0.041 (0.424)	-0.79	0.87
4B	Condition × XG quadratic	-0.164 (0.066)*	-0.29	-0.03
4B	Expected fullness intensity	-12.70 (1.18)***	-15.01	-10.39
4B	Perceived sweetness intensity	-1.90 (1.05)	-3.96	0.16
4B	Perceived viscosity intensity	-7.02 (1.02)***	-9.02	-5.02

Note. β (SE) and 95% CI for all fixed effects across Models 4U, 4A, and 4B (see Table 7 for model specifications and random-effect variances). *p<0.05, **p<0.01, ***p<0.001.

Supplementary Table S5. Ordinal-logit robustness check for intake, compared with the continuous LMM (Model 4U)

Term	Ordered logit β (SE)	p	Consistent with LMM (4U)
XG Linear	-0.191 (0.018)	<0.001	Yes (negative, significant)
XG quadratic	0.0073 (0.0030)	0.017	Yes (positive, significant)
Informed (condition)	1.012 (0.191)	<0.001	Yes (positive, significant)
Interactions			
Condition × XG linear	-0.010 (0.023)	0.678	Yes (n.s. in both)
Condition x XG quadratic	-0.008 (0.004)	0.057	Bordeline (sig. in LMM)

Note. Random-intercept ordered logistic regression treating intake as five-level ordered response. Coefficients are on the log-odds scale and are not directly comparable in magnitude with the LMM; the sign and significance of the principal XG effects match model 4U, confirming that the main conclusions are not sensitive to the continuous approximation. Cut points (not shown): -3.14, -0.68, 1.37, 3.57).

Supplementary Table S6. Curvature of the sensory-to-liking relationships, with vertex location

Term	Texture – Viscosity	Taste – Sweetness
XG Linear	-0.310 (0.031)***	-0.286 (0.043)***
XG quadratic	-0.041 (0.010)***	-0.103 (0.015)***
Informed (condition)	0.263 (0.086)**	0.291 (0.082)***
Interactions		
Condition × XG linear	0.041 (0.041)	-0.053 (0.055)
Vertex (centered)	-3.81 (-5.73, -1.89)	-1.39 (-1.92, -0.86)
Observed range (centered)	-4.36 to 3.64	-5.28 to 2.72

Supplementary Table S7. Random effects variance components for Table 4.

Attribute	Var(intercept)	Var(XG slope)	Var(residual)
Perceived viscosity	1.153	0.0005	2.059
Perceived sweetness	0.944	0.0012	1.416
Perceived satiety	1.367	0.0022	1.412
Texture liking	1.746	0.0077	1.776
Taste liking	1.855	0.0036	1.708
Viscosity liking	1.599	0.0126	1.893
Overall liking	1.598	0.0066	1.312
